# Supplementary material for: Characterization of RARRES1 Expression on Circulating Tumor Cells as Unfavorable Prognostic Marker in Resected Pancreatic Ductal Adenocarcinoma Patients
Source: Cancers (Basel). 2022 Sep 10;14(18):4405. doi: 10.3390/cancers14184405 (PMC9497091; doi:10.3390/cancers14184405)
Supplement: Supplementary file 1 [file cancers-14-04405-s001.zip › Supplementary Table S2.pdf]

Supplementary Table S2: Correlation of CTC detection at baseline with clinicopathological parameters in curative and palliative patients

A. Curative patients

		Curative patients n=36		No CTC detection at baseline n=27		CTC detection at baseline n=9		P value
		n	%	n	%	n	%	
Age	≤ 67 years	18	50.0	14	51.9	4	44.4	
	> 67 years	18	50.0	13	48.1	5	55.6	1.000
Gender	male	17	47.2	13	48.1	4	44.4	
	female	19	52.8	14	51.9	5	55.6	1.000
ECOG	0	20	55.6	16	59.3	4	44.4	
	1	14	38.9	9	33.3	5	55.6	
	2	2	5.6	2	7.4	0	0	0.565
Neoadjuvant treatment	no	29	80.6	22	81.5	7	77.8	
	yes	7	19.4	5	18.5	2	22.2	1.000
Surgical procedure	PD/PPPD	20	55.6	15	55.6	5	55.6	
	left pancreatectomy	13	36.1	10	37.0	3	33.3	
	total pancreatectomy	3	8.3	2	7.4	1	11.1	1.000
Adjuvant treatment	yes	7	19.4	6	22.2	1	11.1	
	no[1]	29	80.6	21	77.8	8	88.9	0.652
Dindo classification	0-2	19	52.8	15	55.6	4	44.4	
	3-4	12	33.3	7	25.9	5	55.6	
	5	5	13.9	5	18.5	0	0	0.386
pT stage	T1-2	17	47.2	13	48.1	4	44.4	
	T3-4	19	52.8	14	51.9	5	55.6	1.000
pN stage	N0	10	27.8	9	33.3	1	11.1	
	N+ (N1/2)	26	72.2	18	66.7	8	88.9	0.392
Grading[2]	G2	23	69.7	16	66.7	7	77.8	
	G3	10	30.3	8	33.3	2	22.2	0.686
R status	R0, CRM-	18	50.0	14	51.9	4	44.4	
	R0, CRM+ / R1	18	50.0	13	48.1	5	55.6	1.000
UICC	I-II	28	77.8	22	81.5	6	66.7	
	III	8	22.2	5	18.5	3	33.3	0.384
Ca 19-9	≤ 500U/ml	26	72.2	19	70.4	7	77.8	
	> 500U/ml	10	27.8	8	29.6	2	22.2	1.000
Recurrence	yes	16	44.4	14	51.9	2	22.2	
	no	20	55.6	13	48.1	7	77.8	0.245

[1] Not started during follow-up period, or due to reduced ECOG or death
[2] For n=3 patients no grading (G) is available

B. Palliative patients

		Palliative patients n=19						
				No CTC detection at baseline n=14		CTC detection at baseline n=5		P value
		n	%	n	%	n	%	
Age	≤ 67 years	9	47.4	5	35.7	4	80.0	
	> 67 years	10	52.6	9	64.3	1	20.0	0.141
Gender	male	14	73.7	10	71.4	4	80.0	
	female	5	26.3	4	28.6	1	20.0	1.000
ECOG	0	5	26.3	3	21.4	2	40.0	
	1	11	57.9	8	57.1	3	60.0	
	2	3	15.8	3	21.4	0	0	0.787
UICC	III	3	15.8	3	21.4	0	0	
	IV	16	84.2	11	78.6	5	100.0	0.530
Ca 19-9	≤ 500U/ml	7	36.8	7	50.0	0	0	
	> 500U/ml	12	63.2	7	50.0	5	100.0	0.106

ctDNA, circulating tumor DNA; ECOG, Eastern Cooperative Oncology Group; CRM, circumferential resection margin; Ca 19-9, Carbohydrate Antigen 19-9; UICC, Union for International Cancer Control; PD, partial pancreatoduodenectomy; PPPD, pylorus preserving pancreatoduodenectomy
