# Supplementary figures and images for: Characterization of RARRES1 Expression on Circulating Tumor Cells as Unfavorable Prognostic Marker in Resected Pancreatic Ductal Adenocarcinoma Patients
Source: Cancers (Basel). 2022 Sep 10;14(18):4405. doi: 10.3390/cancers14184405 (PMC9497091; doi:10.3390/cancers14184405)

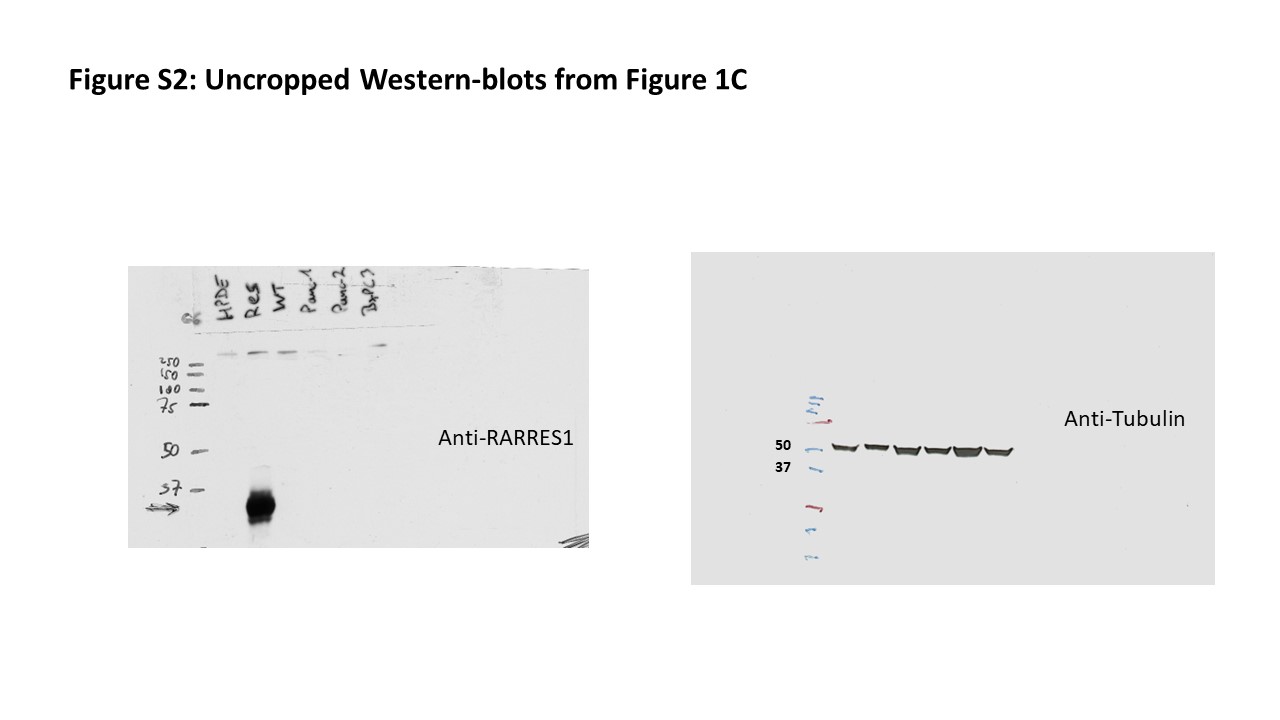

Supplement: Supplementary file 1 [file cancers-14-04405-s001.zip › Suppl.Figure S2 .jpg]

### Supplementary Figure S1: Study cohort

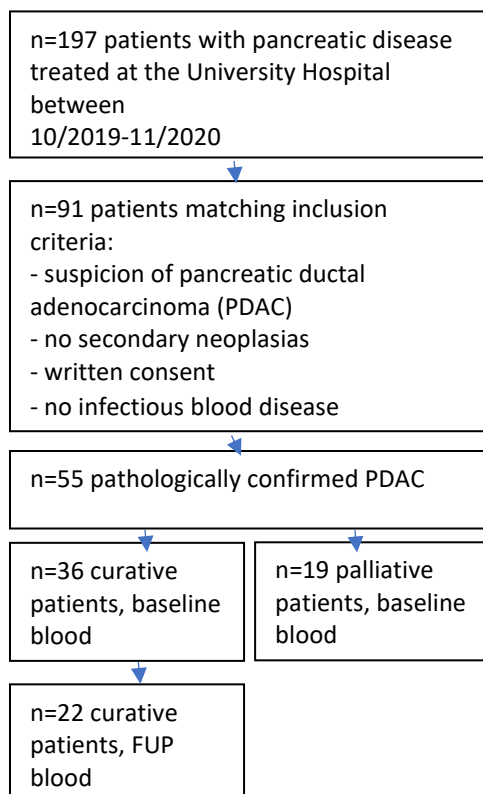

**Figure S1.** Study cohort recruitment flowchart.

Supplement: Supplementary file 1 [file cancers-14-04405-s001.zip › Supplementary figure S1.pdf]
